# First Complete Genome Sequence of *Cherry virus A*

**DOI:** 10.1128/genomeA.00498-16

**Published:** 2016-06-09

**Authors:** Hiroaki Koinuma, Takamichi Nijo, Nozomu Iwabuchi, Tetsuya Yoshida, Takuya Keima, Yukari Okano, Kensaku Maejima, Yasuyuki Yamaji, Shigetou Namba

**Affiliations:** Department of Agricultural and Environmental Biology, Graduate School of Agricultural and Life Sciences, The University of Tokyo, Tokyo, Japan

## Abstract

The 5′-terminal genomic sequence of *Cherry virus A* (CVA) has long been unknown. We determined the first complete genome sequence of an apricot isolate of CVA (7,434 nucleotides [nt]). The 5′-untranslated region was 107 nt in length, which was 53 nt longer than those of known CVA sequences.

## GENOME ANNOUNCEMENT

*Cherry virus A* (CVA) is a member of the genus *Capillovirus* in the family *Betaflexiviridae*. CVA possesses flexuous filamentous particles containing a monopartite, single-stranded, and positive-sense RNA genome with two open reading frames (ORFs). Although the draft genome sequence (7,383 nucleotides [nt]) of CVA was first reported in 1995 (type isolate, GenBank accession no. X82547), its 5′ end was not determined (1).

In 2015, leaves of an apricot (*Prunus armeniaca* cv. Heiwamaru) showing symptoms of vein clearing were collected in Tokyo, Japan. Total RNA was extracted from leaves using the RNeasy plant minikit (Qiagen, Germany). The cDNA library was constructed using the TruSeq RNA sample prep kit version 2 (Illumina, USA), and the library was paired-end sequenced by MiSeq (Illumina) with reagent kit version 2 (500 cycles). The reads were *de novo* assembled using the Oases program (2), and the assembled contigs were subjected to BLASTn search (3) against the GenBank database. A single contig (7,408 nt) showing 86% sequence identity with the CVA type isolate was obtained, and no other contigs showed sequence similarity with viruses/viroids. The 5′ end of the genome was amplified by rapid amplification of cDNA ends (RACE) using the Gene Racer kit (Invitrogen, USA). The 3′ end of the genome was amplified by reverse transcription-PCR (RT-PCR) using CVA-specific and oligo(dT) primers. The amplified fragments were cloned into pCR-Blunt II-TOPO vector (Invitrogen), after which 6 and 8 clones of the 5′ and 3′ ends of the genome were sequenced, respectively.

The complete genome sequence of the apricot isolate of CVA, designated CVA-J, was 7,434 nt long, excluding the poly(A) tail at its 3′ end. A comparative analysis between CVA-J and other isolates of CVA was conducted using the SDT program (4) based on pairwise alignments using the MUSCLE algorithm (5). ORF1 (nt 108 to 7136) of CVA-J shared 81.2 to 98.0% nt identity and 85.0 to 98.4% amino acid (aa) identity with five other CVA isolates. ORF2 (nt 5453 to 6844) of CVA-J shared 90.6 to 98.8% nt identity and 88.1 to 97.8% aa identity with 11 other CVA isolates. Two isolates from noncherry hosts (accession numbers LN879388 and HQ267856) showed high nucleotide sequence identity (ORF1, 98.0%; ORF2, 98.6 to 98.8%) with CVA-J, while isolates from sweet-and-sour cherries showed low nucleotide sequence identity (ORF1, 81.2 to 85.9%; ORF2, 90.6 to 92.9%) with CVA-J, in accordance with a previous report (6).

The length of the 5′ untranslated region (UTR) of CVA-J was 107 nt, which was 53 nt longer than those of known CVA sequences. This is probably because these earlier sequences were determined using forward primers designed on the uncompleted 5′-terminal sequence of the type isolate (7). The 5′-terminal nucleotide of CVA-J was G, which is observed in many viruses in the family *Betaflexiviridae* and *Alphaflexiviridae*. For flexiviruses (to which both of these families belong), G at the 5′ end is believed to serve as an efficient transcription initiation site of genomic and subgenomic RNAs (8).

In this study, we determined for the first time the complete genome sequence of CVA. Our results will be helpful for further investigations of the sequence diversity of CVA.

### Nucleotide sequence accession number.

The complete genome sequence of CVA-J has been deposited in the DNA Data Bank of Japan under accession no. LC125634.
